# The Status and Prospects of Epstein–Barr Virus Prophylactic Vaccine Development

**DOI:** 10.3389/fimmu.2021.677027

**Published:** 2021-06-08

**Authors:** Cong Sun, Xin-chun Chen, Yin-feng Kang, Mu-sheng Zeng

**Affiliations:** State Key Laboratory of Oncology in South China, Collaborative Innovation Center for Cancer Medicine, Guangdong Key Laboratory of Nasopharyngeal Carcinoma Diagnosis and Therapy, Department of Experimental Research, Sun Yat-sen University Cancer Center, Sun Yat-sen University, Guangzhou, China

**Keywords:** Epstein–Barr virus, vaccine, virus immunology, adjuvant, animal model

## Abstract

Epstein–Barr virus (EBV) is a human herpesvirus that is common among the global population, causing an enormous disease burden. EBV can directly cause infectious mononucleosis and is also associated with various malignancies and autoimmune diseases. In order to prevent primary infection and subsequent chronic disease, efforts have been made to develop a prophylactic vaccine against EBV in recent years, but there is still no vaccine in clinical use. The outbreak of the COVID-19 pandemic and the global cooperation in vaccine development against SARS-CoV-2 provide insights for next-generation antiviral vaccine design and opportunities for developing an effective prophylactic EBV vaccine. With improvements in antigen selection, vaccine platforms, formulation and evaluation systems, novel vaccines against EBV are expected to elicit dual protection against infection of both B lymphocytes and epithelial cells. This would provide sustainable immunity against EBV-associated malignancies, finally enabling the control of worldwide EBV infection and management of EBV-associated diseases.

## Introduction

Epstein–Barr virus (EBV) is a double-stranded DNA virus that belongs to the gamma herpesvirus family. It causes endemic infection in over 95% of the worldwide population (1), and is associated with diseases such as infectious mononucleosis (IM) and a broad range of lymphoid or epithelial malignancies (2, 3). It is estimated that approximately 2% of malignancies are caused by EBV infection, resulting in over 200,000 cases of EBV-associated cancer each year (4).

The transmission of EBV within the population is mainly mediated by saliva, and the infection involves both B lymphocytes and epithelial cells (5). Primary infection mostly occurs in early childhood with little or no overt symptoms (6). After the primary infection is established, EBV sustains a persistent infection in B lymphocytes, accompanied by the expression of specialized viral genes that maintain its latency, which is associated with B cell tumorigenesis (7). Therefore, a prophylactic EBV vaccine for establishing early protection against primary infection is critical for prevention both infectious diseases and EBV-associated malignancies. However, there is still no prophylactic vaccine against EBV in clinical use due to various reasons including antigen selection, vaccine platform used, and evaluation system for EBV vaccine assessment. Thus, in this review, we summarize the challenges and opportunities encountered in the development of a prophylactic EBV vaccine.

## Antigen Selection for Vaccine Design

### Glycoproteins

**Figure 1:**
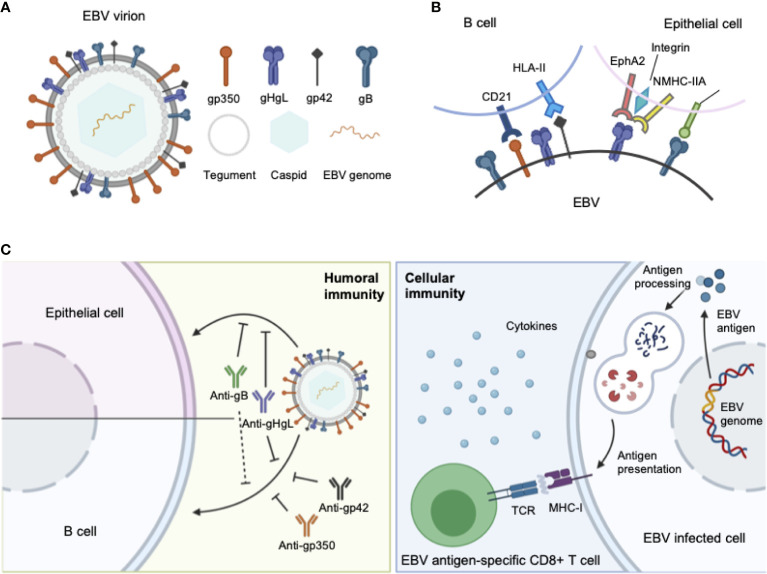
**(A)** Structure of the EBV virion. As an enveloped double-stranded DNA virus, the virion of EBV consists of a lipid membrane, tegument, viral capsid and the packed EBV genome. Glycoproteins are distributed on the virion membrane and are crucial for recognition, host cell attachment and membrane fusion. **(B)** The major interaction pattern of host cell receptors and the EBV membrane glycoproteins. EBV infects B lymphocytes and epithelial cells *via* different combinations of ligand–receptor interaction. **(C)** Humoral and cellular immunity against EBV infection. In humoral immunity, antibodies against various glycoproteins play different roles in the neutralization process. After infection, EBV antigen can be presented, inducing a cytotoxic CD8^+^ T cell response against infected cells.

Similar to other herpesviruses, EBV is an enveloped virus, comprising a membrane decorated with envelope glycoproteins (such as gp350, gp42, gH, gL, and gB), which are crucial for receptor recognition, attachment, and virus–host membrane fusion (8). As EBV can infect both B lymphocytes and epithelial cells, the glycoproteins involved in the infection process of each cell type differ. For B cell infection, EBV gp350 interacts with CD21 or CD35 on B cells to establish viral binding, followed by the binding of gp42 in complex with gHgL to HLA class II on B cells, after which gB eventually triggers the membrane fusion in endocytic vesicles (9–12). By contrast, EBV adopts a rather different and more versatile combination of ligand–receptor paring during viral entry into epithelial cells. EBV can still use gp350 to establish attachment to CD21-expressing host cells (13), while BMRF2 (14) or the gH/gL complex binds to other host cell receptors to facilitate the infection of cells that lack CD21. Currently, integrins (15), non-muscle myosin heavy chain IIA (NMHC-IIA) (16) and ephrin receptor A2 (17, 18) (EphA2) are recognized as receptors for EBV gH/gL, while neuropilin-1 (19) (NRP1) acts as the receptor for EBV gB during epithelial cell infection, indicating a more important role of gH/gL complex and gB during the recognition and attachment in comparison to B cell infection (**Figure 1**).

The complex molecular machine of surface glycoproteins brings challenges for not only understanding the complete fusion mechanism of EBV, but also choosing appropriate antigens for vaccine development. Therefore, an ideal prophylactic vaccine against EBV should be able to elicit potent neutralizing antibodies against EBV infection in both B lymphocytes and epithelial cells, which requires careful selection of antigens during vaccine design.

#### gp350

**Figure 2:**
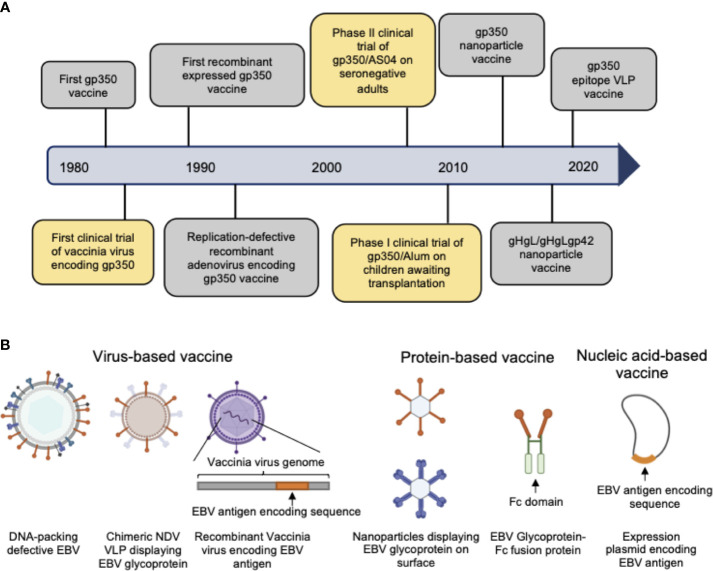
**(A)** Hallmarks of prophylactic EBV vaccine development using EBV glycoproteins as antigens. Clinical trials are marked in yellow box and others are marked in gray box. **(B)** Current candidate platforms for EBV vaccines, including virus-based, protein-based and nucleic-acid-based vaccines.

As the first-isolated and most abundant EBV glycoprotein, gp350 was the most studied antigen for vaccine candidates and is the core antigen for the majority of the currently-developed EBV vaccines (20–28). The first clinical trial of a recombinant viral vector encoding gp350 performed in China in 1997 proved that gp350-specific antibodies could be elicited in both seronegative and seropositive children (29). In later studies, recombinant gp350 adjuvanted with AS04 was used as the vaccine in a phase II clinical trial among seronegative adults (30) and was shown to effectively reduce the incidence of IM compared to the placebo control group. However, this vaccine did not completely prevent EBV infection in the vaccinated population. In another phase I clinical trial gp350 was formulated with 0.2% Alhydrogel^®^ as vaccine for reducing the risk of post-transplant lymphoproliferative disease (PTLD) (31). The vaccine failed to elicit neutralizing antibodies and control the viral titer in the majority of patients, possibly due to its low immunogenicity for immunosuppressed patients. Thus, despite the early and thorough study, gp350 exhibited only imperfect vaccination efficacy as single antigen in both preclinical and clinical trials (**Figure 2A**, **Tables 1**, **2**).

**Table 1:** Summary of EBV vaccine animal trials.

Year	Platform/ Adjuvant	Antigen	Animals	Results
1984	Subunit vaccine/liposome, Freund’s adjuvant, lipid A	Full length membrane gp340(gp350) purified from virus	Mice, rabbit and cottontop tamarins	Antibody responses were induced similarly in mice and cotton-top tamarins, among which groups adjuvanted with liposome and lipid A elicited antibody responses earlier; Antibody responses in rabbits were rather weak (32).
1985	Prototype subunit vaccine	Full length membrane gp340(gp350) purified from virus	cottontop tamarins	Privided protection against malignant lymphoma (21)
1985	Recombinant vaccinia virus(WR strain)	gp340	rabbits	Neutralizing antibodies against gp340 could be detected (22).
1986	Subunit vaccine	gp340 produced by immunoaffinity chromatography from B95-8.	cottontop tamarins	No protection against malignant lymphoma (23)
1988	Subunit vaccine	gp350/gp220 produced by immunoaffinity chromatography from yeast and mammalian cells	——	All of the mammalian cell-derived versions of the membrane antigen were found capable of inducing EBV-specific neutralizing antibodies as well as B95-8 (33).
1988	Subunit vaccine/ ISCOMS	gp340 incorporated into immune-stimulating complexes (ISCOMS)	cottontop tamarins	Provided protection against malignant lymphoma (34)
1988	Recombinant vaccinia virus (Wyeth or WR strains)	gp340	cottontop tamarins	Only WR strain derived vaccine could offer protection against malignant lymphoma (24)
1992	Recombinant subunit vaccine/ threonylmuramyl dipeptide adjuvant formulation.	gp340, lack of membrane anchor region, produced using a bovine papillomavirus (BPV) expression vector	cottontop tamarins	3/4 immunized cottontop tamarins showed protection against malignant lymphoma, 1/4 immunized cottontop tamarins developed idiopathic colitis due to low immune responses to gp340 (35).
1993	Replication-defective recombinant adenovirus vaccine	gp340/220	cottontop tamarins	Provided protection against malignant lymphoma despite no detectable neutralizing antibodies in vitro (25).
1994	Subunit vaccine/alum	gp340	Rabbits, cottontop tamarins	3/5 immunized cottontop tamarins showed protection against malignant lymphoma (36).
1996	Recombinant vaccinia virus	gp340	common marmosets challenged with M81	Vaccinated group showed lower virus load compared to control group (26).
1999	Recombinant subunit vaccine/ alum VS Freund’s adjuvant	Single chain gp350	rabbits	Elicited high neutralizing antibody titers; three immunizations with MSTOP gp350 elicited neutralizing titers of 3800±5400 in alum and 1,600 ± 3,400 in Freund's adjuvant (27).
2001	Peptide epitopes	HLA A2-restricted epitopes from the latent, lytic and structural proteins	Humanized HLA A2/Kb mice	A maximal response to the epitopes within the structural proteins and low to moderate responses to the latent epitopes, indicating hierarchy of CTL responses between mice and humans (37).
2003	Recombinant poxvirus vaccine	Polyepitope protein comprising 6 HLA A2–restricted epitopes derived from LMP1	Humanized HLA A2/Kb mice	Successfully reversed the out- growth of LMP1-expressing tumors in HLA A2/Kb mice (38).
2009	Epitope/HSP70 and incomplete Freund's adjuvant	Mycobacterial HSP70 and LMP2A (356-364) epitope fusion protein	Humanized HLA-A2.1 mice	Specific CTL more effectively than a single peptide plus incomplete Freund's adjuvant; melanoma tumor cells was suppressed in humanized HLA-A2.1 mice (39).
2009	Recombinant adeno-associated virus/HSP	Latent membrane proteins (LMP1 and LMP2) CTL epitope	BALB/c (H-2d) mice	Specific CTL responses; eliminated tumors in mice (40).
2011	Epitope/ HSP70	Reconstituted complexes of MtHsp70 and LMP2A-peptides	HLA-A2.1 transgenic mice	Specific CTL responses; protective activity and therapeutic efficacy against LMP2A-expressed tumor challenge (41).
2011	EBV-derived VLP	EBV-derived VLP, deleted or function- ally inactivated six viral genes (EBNA2, LMP1, EBNA3A, -B, and -C, BZLF1)	BALB/c mice	Strong CD8^+^ and CD4+ T cell responses in a preclinical murine model (42).
2011	Combined immunization of DNA, AAV, and adenovirus vector vaccines	LMP2	BALB/c mice	Combined immunization with DNA, AAV, and adenovirus vector vaccines induced specific cellular immunity better than any other combinations (43).
2013	Multimeric subunit vaccine/tetanus toxoid	gp350 (1-470)	BALB/c mice	Tetrameric gp350 induced ∼20-fold higher serum titers of specific IgG and >19-fold enhancements in neutralizing titers at the highest dose;tetanus toxoid (TT)-specific CD4+ T-cell epitopes into the tetrameric gp350: no effect on specific antibody responses (44).
2013	Replication-defective chimpanzee-derived adenovirus vectors	Rhesus Lymphocryptovirus EBNA-1 Homologue, rhEBNA-1	rhesus macaques	EBNA-1-specific T cells could be expanded by vaccination (45).
2013	Recombinant subunit vaccine	Truncated EBNA1 (E1ΔGA, codons 390–641), produced from methylotrophic yeast P. pastoris	BALB/c mice	Elicited CD4+ and CD8+ T cell responses (46)
2015	Newcastle disease virus (NDV)-virus-like particle	EBV gp350/220 ectodomain	BALB/c mice	Elicited neutralizing antibody responses, but not better than soluble gp350/220 (47).
2015	Dendritic cells pulsed with recombinant BZLF1	BZLF1	hu-PBL-SCID mice	Elicited specific cellular immunity; improved survival from fatal EBV-LPD (48).
2015	Self-assembling nanoparticles	gp350 D_123_-ferritin; gp350 D123- encapsulin	BALB/c mice; Cynomolgus Macaques	gp350-nanoparticle elicited 10- to 100-fold higher neutralization titer compared to soluble gp350 (49).
2015	Recombinant subunit vaccine/ TiterMax (CytRx)	native or denatured/alkylated gp350 produced from CHO	Rabbits	Denatured gp350 could induce binding antibodies but no neutralizing antibodies (28).
2015	Designed peptides, coupled with keyhole limpet hemocyanin (KLH), Sigma adjuvant system	Designed gp350 peptides to mimic gp350 amino terminus that interacts with 72A1	BALB/c mice	The gp350 mimetic peptide bound to 72A1 antibody can block gp350 recognition (50).
2016	Multimeric subunit vaccine	trimeric gH/gL; trimeric gB; tetrameric gp350	rabbits	Trimeric and monomeric gH/gL, trimeric gB, and tetrameric gp350 groups induced serum EBV-neutralizing titers >100-, 20-, 18-, and 4-fold higher, respectively, than monomeric gp350 (51).
2016	Multi-epitope vaccine	Chimeric multi-epitope protein referred to as EBV-LMP2m, which is composed of LMP2aa195-232 and LMP2aa419-436	BALB/c mice	Elicited specific antibody and CTL responses (52)
2018	Subunit vaccine	Fc-fused gp350 dimer	BALB/c mice	Elicited higher specific antibody titers than gp350 monomer; elicited potent nAbs (53).
2018	EBV-derived VLP	Viral particle expressed both with lytic and latent proteins by insertion of latent protein epitopes into the major tegument protein BNRF1	Humanized NSG-A2 mice	Provide significant protection against wild-type EBV infection (54)
2019	Self-assembling nanoparticles/ SAS adjuvant	gH/gL-ferritin; gH/gL/gp42-ferritin	BALB/c mice; Cynomolgus macaques	Monkey immunized with gH/gL/gp42-ferritin nanoparticles elicited >40- and ~4-fold higher neutralization titers in B cells in comparison with soluble gH/gL and soluble gH/gL/gp42; in epithelial cells, gH/gL-ferritin and gH/ gL/gp42-ferritin nanoparticles showed >25- and ~4-fold higher neutralizing titers than the corresponding soluble glycoprotein vaccines (55).
2020	Newcastle disease virus (NDV)-virus-like particle/ aluminum hydroxide and monophosphoryl lipid A	gp350, gB, gp42, gH, and gL pentavalent complex	rabbits	Elicited specific neutralizing antibodies more robust than soluble gp350 ectodomain (56).
2020	Epitope VLP	Combinations of three gp350 epitopes from receptor-binding domain (aa 16–29/ aa 142–161/ aa 282-301)	BALB/c mice	elicited neutralizing antibodies (57)

**Table 2:** Summary of EBV vaccine clinical trials.

Trial ID	Published Year	Phase	Platform/ Adjuvant	Antigen	Subjects	Observation index	Results
——	1995	——	Recombinant vaccinia virus	Major EBV membrane antigen BNLF-1 MA (gp 220–340)	EBV-positive and vaccinia-virus-exposed adults; EBV-positive, non-vaccinia-virus-exposed juveniles; and EBV and vaccinia virus-naive infants	EBV infection status for EBV negative infants	EBV-neutralizing titers increased in the vaccinated juveniles compared to adults; 9/9 vaccinated infants had specific neutralizing antibody response and only three of them vaccinated infants infected EBV while 10/10 unvaccinated infants got infected (29).
——	2002	I	Epstein–Barr Virus (EBV) Peptide-pulsed Dendritic Cells	LMP2	Patients with advanced NPC	Clinical responses in 1-year follow-up: PR, partial response; PD, progressive disease	9/16 patients had epitope-specific CTL responses; 2/16 patients had lesions shrunk (58).
——	2007	I	Subunit vaccine/AS04	gp350	Healthy adults (EBV + and EBV− both included)	Incidence of infectious mononucleosis	Seroconversion rates were 100%; adjuvanted gp350 vaccine is better than non-adjuvanted in terms of GMTs for anti-gp350 ELISA responses (20).
——	2007	I/II	Subunit vaccine/AS04	gp350	EBV-seronegative subjects	Incidence of infectious mononucleosis
NCT00430534	2007	II	Subunit vaccine/AS04	gp350	EBV-seronegative healthy Young Adults	Incidence of infectious mononucleosis	78.0% efficacy in preventing IM, no efficacy in preventing asymptomatic EBV infection; 98.7% showed seroconversion to anti-gp350 antibodies, remained anti-gp350 antibody positive for >18 months (30).
——	2008	I	CD8+ T-Cell peptide epitope-Based vaccine/fusion with tetanus toxoid formulated in a water-in-oil adjuvant, Montanide ISA 720	HLA B*0801-restricted peptide epitope FLRGRAYGL from EBNA3 and tetanus toxoid	Healthy EBV-seronegative 18- to 50-year-old individuals	Incidence of infectious mononucleosis	epitope-specific responses were detected in 8/9 peptide-vaccine recipients and 0/4 placebo vaccine recipients; 1/2 placebo vaccinees who acquired EBV developed infectious mononucleosis, whereas 4/4 vaccinees who acquired EBV after completing peptide vaccination seroconverted asymptomatically (59).
——	2009	I	Subunit vaccine /alhydrogel	gp350	Children with chronic kidney disease awaiting transplantation	Incidence of lymphoproliferative disease after transplantation	Neutralizing antibodies were detected in four recipients (1/4 in the 12.5 ug and 3/9 in the 25 ug cohort) (31).
——	2012	II	Adenovirus-△LMP1-LMP2 transduced dendritic cell	a truncated LMP1 (△LMP1, inactive form) and full-length LMP2	EBV-positive metastatic NPC (World Health Organization type II/III)	Clinical responses in 14-weeks follow-up: complete response (CR), partial response (PR) and stable disease (SD)—of longer than 14 weeks	DCs activated LMP1/2-specific T cells in vitro, no such increase in the frequency of peripheral LMP1/2-specific T cells was detected. Three patients had clinical responses including one with partial response (for 7.5 months) and two with stable disease (for 6.5 and 7.5 months) (60).
NCT01256853	2013	I	Recombinant modified vaccinia Ankara	EBNA1/LMP2 fusion protein	NPC patients, Clinically, all in remission more than 12 weeks after primary therapy	Frequency of functional T-cell responses; levels of EBV genomes in plasma (to reflect tumor burden).	T-cell responses to one or both vaccine antigens were increased in 15 of 18 patients (61).
NCT01147991	2014	I	Recombinant modified vaccinia Ankara	EBNA1 and LMP2	EBV-Positive NPC	——	T-cell response rates: 7/14 for EBNA1; 6/14 for LMP2 (62).

#### gH/gL

Since the gH/gL complex plays a critical role in the infection of B cells and especially epithelial cells, there is increasing focus on the gH/gL complex as the antigen for new vaccine candidates. The recently identified anti-gH/gL dual-tropic neutralizing antibody AMMO1 (63) further indicated that gH/gL may be an ideal antigen. In a study on rabbits (51), trimeric or monomeric gH/gL could elicit >100- and 18-fold higher EBV neutralizing antibody titers than monomeric gp350. Later nanoparticle vaccines displaying gH/gL or the gH/gL/gp42 complex were designed, and immunization assays in BALB/c mice demonstrated that the nanoparticle decorated with gH/gL or gH/gL/gp42 could elicit much higher neutralizing antibody titers than monomeric gH/gL or gH/gL/gp42 (55). Despite these promising results for gH/gL as a vaccine candidate, there are still no clinical trials examining whether gH/gL could provide broader protection than gp350 and possibly achieve complete protection from EBV infection.

#### gp42

gp42 is a subunit of the gH/gL/gp42 heterotrimer on the EBV virion membrane. It was identified as the ligand for HLA class II molecules mostly participating in B cell infection and was recently found to hinder the infection of epithelial cells (64, 65), indicating that it controls the tropism of EBV infection. The close structural connection and functional complexity suggested that a combination of gH/gL/gp42 as a complex antigen may be more potent than gp42 alone. Studies of the effect of immunization with the EBV viral fusion apparatus indicated that immunization using gH/gL in complex with gp42, either as monomers or nanoparticles, could elicit relatively higher neutralizing antibody titers against infection of both B lymphocytes and epithelial cells (55). Nevertheless, few studies investigated gp42 as the target for vaccine design. Moreover, its role in controlling the tropism of infection would complicate the protection efficacy of elicited antibodies against gp42, which may potentially influence the tropism of the original virus and enhance the efficiency of epithelial cell infection.

#### gB

EBV gB is the fusion protein on the viral surface mediating viral–host membrane fusion and recognizing NRP1 on epithelial cells. Most prophylactic antiviral vaccines target the viral fusion protein, such as influenza HA (66–68), HIV env (69, 70), Ebola virus GP (71, 72), and coronavirus spike protein (73, 74), as the fusion proteins in these viruses not only drive membrane fusion but also recognize host membrane factors to initiate attachment and trigger the fusion process. Thus, in these viruses the functional domain of the fusion protein is considered an ideal vulnerable site for neutralization. Hence, the similarity between EBV gB and other comprehensively studied viral fusion proteins indicates that gB could be a promising target for vaccine development. In addition to the AMMO1 antibody targeting EBV gHgL, anti-EBV gB AMMO2/3/4/5 discovered by ﻿Snijder et al. also demonstrated a strong neutralization activity against epithelial cell infection (63), supporting the use of gB as a prophylactic vaccine candidate. In addition, the previously mentioned research studying the efficacy of immunization with trimeric gH/gL in rabbits also explored gB, which was also able to elicit higher neutralizing antibody titers than gp350 (51).

With the development of protein structure analysis, the fusion status of fusion proteins becomes increasingly important for elucidating the fusion mechanism and understanding the connection between conformational changes and the fusion process. Pre-fusion status is often regarded as the natural conformation on the viral membrane (75, 76) before interacting with the host cell. The discovery of pre-fusion status and artificial modification to freeze the fusion protein in the pre-fusion conformation (77–79) greatly promoted vaccine development in recent years. During the SARS-CoV-2 pandemic, the pre-fusion-stabilized spike protein variant S-2P (80, 81) provided an ideal antigen for the design of broad-use COVID-19 vaccines. Similarly, pre-fusion-stabilized HIV env BG505-SOSIP (69) and RSV F DS-CAV1 (77, 79) also provided an impulse for vaccine development, since they could elicit much higher neutralizing antibody titers than the post-fusion conformation. Therefore, there is increasing focus on the conformation of EBV gB. However, the currently available crystal structure of gB shows a post-fusion conformation at pH8.0 (82), and there is still no high-resolution structure of any pre-fusion gB from the herpesvirus family. Although recent cryo-electron imaging studies of gB displayed on vesicles (83), pseudo-virus membranes or virions (84) were highly suggestive of a potential pre-fusion conformation of gB from other herpesviruses; more evidence and structural studies are required to define the pre-fusion form of gB from EBV, which would greatly promote the use of this antigen as a vaccine candidate.

### Latent and Lytic Phase Proteins

After primary infection, EBV undergoes a short period of replication in the oropharynx, after which further infection of B cells ensues, during which glycoproteins encoded by the EBV genome become eclipsed by certain lytic and latent genes, which drive the B cell transformation and latency as summarized by a review (85). Therefore, neutralizing antibodies against glycoproteins cannot induce the clearance of latently infected cells which do not express the target, while T cell-mediated immunity would be critical for controlling EBV infection during pre-latency and latency. With a deeper understanding of the role of T cell immunity in the control of EBV infection and extensive mapping of immuno-focused T cell epitopes of EBV antigens (86–97), the application of latent or lytic phase proteins as vaccine antigens has become a topic of continuing study. Elliott et al. used the EBNA3 HLA-B8 T cell epitope FLRGRAYGL, adjuvanted with tetanus toxoid and Montanide ISA 720, as a vaccine in a phase I trial among EBV sero-negative adults (59). The results showed that despite good vaccine tolerance and reduced incidence of infectious mononucleosis, the vaccination did not protect the subjects from EBV infection. Other CTL epitopes based on LMP1 and LMP2A showed great potential in tumor treatment in preclinical studies (38–41, 52), but none displayed a clear viability as effective antigens to prevent primary infection. Thus, for prophylactic vaccine development, latent or lytic phase proteins could be used as auxiliary boosters for inducing adequate T cell responses, while the major glycoprotein antigens still play the key role in the prevention of primary infection.

Hence, during EBV vaccine development, rational and careful antigen selection is necessary to ensure both robust and comprehensive immunity against EBV infection. There is still a lot of space for extensive study on immunization efficacy of single glycoproteins, especially gH/gL or gB. Additionally, combinatorial use of multiple antigens as vaccine candidates, including glycoprotein sets or glycoprotein-latency protein combinations, deserves further study for eliciting both sufficient neutralizing antibody titers and T cell responses.

## Vaccine Delivery Platforms and Formulations

### Delivery Platform for Vaccine Design

The outbreak of the COVID-19 pandemic has brought significant challenges for global vaccine development, prompting a continuous stream of innovative designs of candidate vaccines against SARS-CoV-2, and thus giving great impetus to next-generation vaccine development. The rapid application of the first clinically used mRNA vaccine developed by Moderna and BioNTech (98, 99) achieved great success in combating SARS-CoV-2 and demonstrated that innovation of new vaccine designs could accelerate the procedures of vaccine development, provide more flexible platforms for antigen delivery, and improve immunization efficacy. Nevertheless, traditional platforms for vaccine development, such as weakened or inactivated virus (100–102), still account for the majority of currently available vaccines and have demonstrated their value during the COVID-19 pandemic due to their outstanding stability, immunogenicity, and convenience in distribution. Therefore, a wider array of adequate platforms for vaccine design is also critical for EBV vaccine development (**Figure 2B**).

#### Virus-Based Vaccines

Because EBV tends to establish a latent infection of host cells, a general approach to induce EBV replication and cell lysis requires complicated procedures and results in a low yield of live virus. Consequently, the development of attenuated virus or inactivated virus vaccines based on authentic EBV is challenging due to limited viral material. Thus, there are few reports on the use of inactivated or attenuated EBV as vaccine candidates. Alternatively, modification of EBV the genome for direct production of defective virions without genomic DNA could be a viable approach for EBV-derived vaccine development. EBV-derived virus-like particles (VLPs) are based on different EBV mutants with various deletions of sets of oncogenic genes or DNA packaging genes (103), produced by inducing cell lines to enter the lytic phase, followed by purification from cell supernatants by centrifugation. Multiple studies developed several EBV VLPs (delta BFLF1/BFRF1, delta BBRF1, delta BFLF2, delta TR terminal repeats) (42, 104–107) by deletion of certain critical genes to obstruct virus replication and DNA packaging. However, the possibility of repacking of EBV DNA would bring safety concerns to such designs. In addition to the construction of VLPs based on EBV itself, a Newcastle disease virus-like particle (ND VLP) platform was also used for the presentation of EBV antigens such as gp350/gp220, combinations of gHgL-EBNA1 or gB/LMP2, and even pentavalent gp350/gH/gL/gp42/gB (47, 56). It may be easier to produce VLPs by additional co-transfection of NDV-F for particle assembly, benefiting the rapid development of safe VLP vaccines.

Another approach for the development of virus-based vaccines is using viral vectors as carriers to deliver targeted antigens by generating recombinant vaccinia virus. After inserting specific sequences encoding EBV antigens into the genome of vaccinia virus, the recombinant virus can infect host cells and drive the expression of exogenous antigen in the cells, leading to antigen processing and presentation *via* the classic MHC-I pathway and activation of antigen-specific cytotoxic CD8^+^ T cells (108, 109). In addition, through maintaining the certain degree of replication function, attenuated self-replicated vaccinia virus could stimulate an even higher immune response than replication-defective virus. The vaccina virus also acted as a self-adjuvant by expressing a broad range of pathogen-associated molecular patterns (PAMPs), increasing the whole immunogenicity of vaccine. Currently, modified vaccinia virus Ankara (MVA) (61, 62, 110, 111), adenovirus (ADV) (60, 112, 113) and Varicella-zoster virus (VZV) (114) have been used as a vector to generate an EBV antigen-carrying recombinant live virus vaccine. However, this technology was more commonly used for developing therapeutic vaccines for the treatment of EBV-associated tumors due to the favorable stimulation of cellular immunity, while few trials investigated its use in prophylactic vaccines since the first human test using gp350 as antigen and smallpox-based vaccinia virus as viral vector (29) due to the uncertain safety and reported adverse events of this platform (115).

#### Protein-Based Vaccines

With the rapid development and great progress in structure-guided protein modification and design (116–122), recombinant proteins have gradually become an effective approach for accurate antigen immunization. As gp350 was firstly applied as antigen for EBV vaccine design, gp350 modification to promote immunization efficacy was also a focus during the early exploration of protein-based vaccines against EBV. In the late 20^th^ century, soluble gp350 protein was successfully expressed as a vaccine antigen (33). Subsequent attempts to enhance the immunogenicity and improve the immunization efficacy aimed to increase the valency or target the protein to antigen presenting cells (APCs) using a variety of methods such as multimerization, nanoparticle assembly and fusion-protein design. For multivalency, tetrameric gp350 was designed by fusing two separate gp350 (1–470) to a C-terminal leucine-zipper with or without T cell epitopes, and the results showed that tetrameric gp350 could elicit higher neutralizing antibody titers than monomeric gp350 (51). Additionally, by fusing gp350 to ferritin or encapsulin, multivalent gp350 nanoparticles (49) were generated and immunization of mice or monkeys showed that nanoparticles elicited much higher neutralizing antibody titers than soluble monomeric gp350. Further, virus challenge experiments also demonstrated that gp350 nanoparticles provide better protection against EBV infection and improve the survival of challenged monkeys. In an effort to both increase the valency and enable APC-targeting, gp350 was fused with the Fc domain of mouse IgG2a (53, 123), rendering a dimeric antibody-like antigen which could target Fc*γ*R on antigen-presenting cells to prolong the retention time for recognition. In addition, the fused protein simplified the purification and detection.

Comparatively few studies investigated using other glycoproteins or latent phase proteins as antigens. Trimeric gHgL constructed by fusing gHgL to a C-terminal trimeric T4 bacteriophage fibritin and native trimeric gB were also tested as immunogens (51), and the results showed that trimeric gHgL could elicit higher neutralizing antibody titers than monomeric gHgL. Recently, analogous methodology was adopted to design gHgL or gHgL/gp42 nanoparticles by fusing the antigen to the 24-mer ferritin, whereby the neutralizing antibody titers of the nanoparticle-immunized groups were significantly higher than in the monomer groups as previously mentioned.

Instead of using the full length or a major segment of the protein, some studies attempted to use specific epitopes as antigens to induce site-specific immune responses and thereby achieve accurate immunization. Jerome et al. designed two 72A1-gp350 blocking peptides that mimic the interacting region of gp350 (50), which demonstrated that the neutralization epitope of the glycoprotein could be an ideal vaccine antigen. Afterwards, Zhang et al. inserted different tandem gp350 epitopes into HBC149 to construct a gp350 epitope-displaying VLP (57), and the neutralizing antibody titers of some gp350 epitope-VLP groups were even higher than that of gp350ECD123, a shortened version of the gp350 ectodomain, which compares favorably to the anti-gp350 nAb 72A1.

#### Nucleic-Acid Vaccines

The rapid and successful application of nucleic-acid SARS-CoV-2 vaccines demonstrated their great potential in viral vaccine development. This method, based on synthetic nucleic acids, enables large-scale manufacturing with almost perfect uniformity. Despite such advantages, the use of synthetic nucleic acids for EBV vaccine development is still in the early exploration phase. Krzysztof et al. developed DNA vaccines based on three EBV latency genes (EBNA1, LMP1 and LMP2A) (124) and found that the vaccine based on EBNA1 and LMP2A could elicit robust T cell immunity. Although mRNA vaccines are highly potent and can be rapidly manufactured, the development of an mRNA vaccine for EBV still awaits the first step. As expected, Moderna has announced its great ambitions in EBV mRNA vaccine development, with the candidate mRNA-1189 encoding all the major glycoproteins (gp350, gB, gH/gL, gp42).

### Adjuvants for Vaccine Formulation

Adjuvants incorporated in components of the antigen for vaccine formulation can modulate the immune response. In addition to the original immunogenic profile of the selected antigen, a carefully selected adjuvant can broaden the use or enhance the efficacy for immunization. For vaccine platforms such as inactivated virus or protein-based subunit vaccines/VLPs, the loss of bioactivity greatly diminishes the immunogenicity of the antigen itself, which further requires powerful adjuvants for pre-stimulation of immune recognition, prolongation of antigen retention, as well as both humoral and cellular immunity enhancement (125–129).

The development of gp350-based vaccines inspired the exploration of adequate adjuvants for EBV vaccines. In the late 20^th^ century, adjuvants such as Freund’s adjuvant, lipid A, immune-stimulating complexes (ISCOMS), and aluminum hydroxide (32, 34–36, 130) were used in the formulation of gp350 vaccines. Some may show superior immunization efficacy compared to unadjuvanted gp350 as immunogen, since an immunization trial of unadjuvanted gp350 subunit vaccine on cotton-top tamarins gave unsatisfactory results, with no protection against incidence of malignant lymphoma in spite of eliciting antibodies against gp350. With the use of more complicated adjuvant systems in recent years, a higher immunization efficacy achieved in preclinical studies supports the case for further clinical trials. However, due to the paucity of studies on other protein-based immunogens as vaccines against EBV, only a limited number of adjuvants were tested. For example, the VZV gE-based vaccine (Shingrix) was the first clinically approved herpesvirus vaccine providing protection against herpes zoster in older adults and immunosuppressed patients, while containing only VZV glycoprotein gE adjuvanted with AS01b (131). Although VZV gE was not used as a prophylactic vaccine antigen to prevent VZV infection, an appropriate combination with the adjuvant made gE into an ideal antigen (132), with benefits for controlling latent VZV infection. This result was based on a systematic screening of appropriate adjuvant systems (133). This study also offers insights for EBV vaccine development, confirming that smart selection of adjuvants can also contribute to the development of a powerful vaccine against EBV by enhancing both initial protection from primary infection and secondary protection from reactivation or expansion of latent infection.

Therefore, an appropriate platform and adjuvant systems also determine the immunization efficacy of the vaccine, and not just the antigen. The COVID-19 pandemic exemplifies the effective and rapid development of vaccines against broadly distributed infectious pathogens. Both mature, extensively tested technologies like inactivated virus (100) and emerging technologies like mRNA vaccines (98, 99) gave satisfactory results, demonstrating the unlimited opportunities of the available vaccine design platforms and encouraging further comparative studies on the use of a variety of platforms for EBV vaccine development. For virus-based vaccines, breakthroughs in the mass production of live EBV could be a solution for inactivated vaccine development, since the latency-preference and complicated induction procedures seriously hinder its manufacture. For the emerging protein- or nucleic acid-based vaccines, convenient modification of antigens to strengthen their immunogenicity and viable co-valency of multiple antigens to broaden the immune response spectrum are promising future approaches for vaccine development. Since the licensed VZV vaccine took the first step in clinical herpes virus immunization, it has brought home the lesson that appropriate adjuvants used in vaccine formulation can greatly enhance the immunization efficacy. Additionally, the rising application of specific toll-like receptor (TLR) agonists (134–136) provides additional alternatives in the selection of adjuvants to achieve specific immunization responses.

## Evaluation Systems for Vaccines

### Animal Models

**Figure 3:**
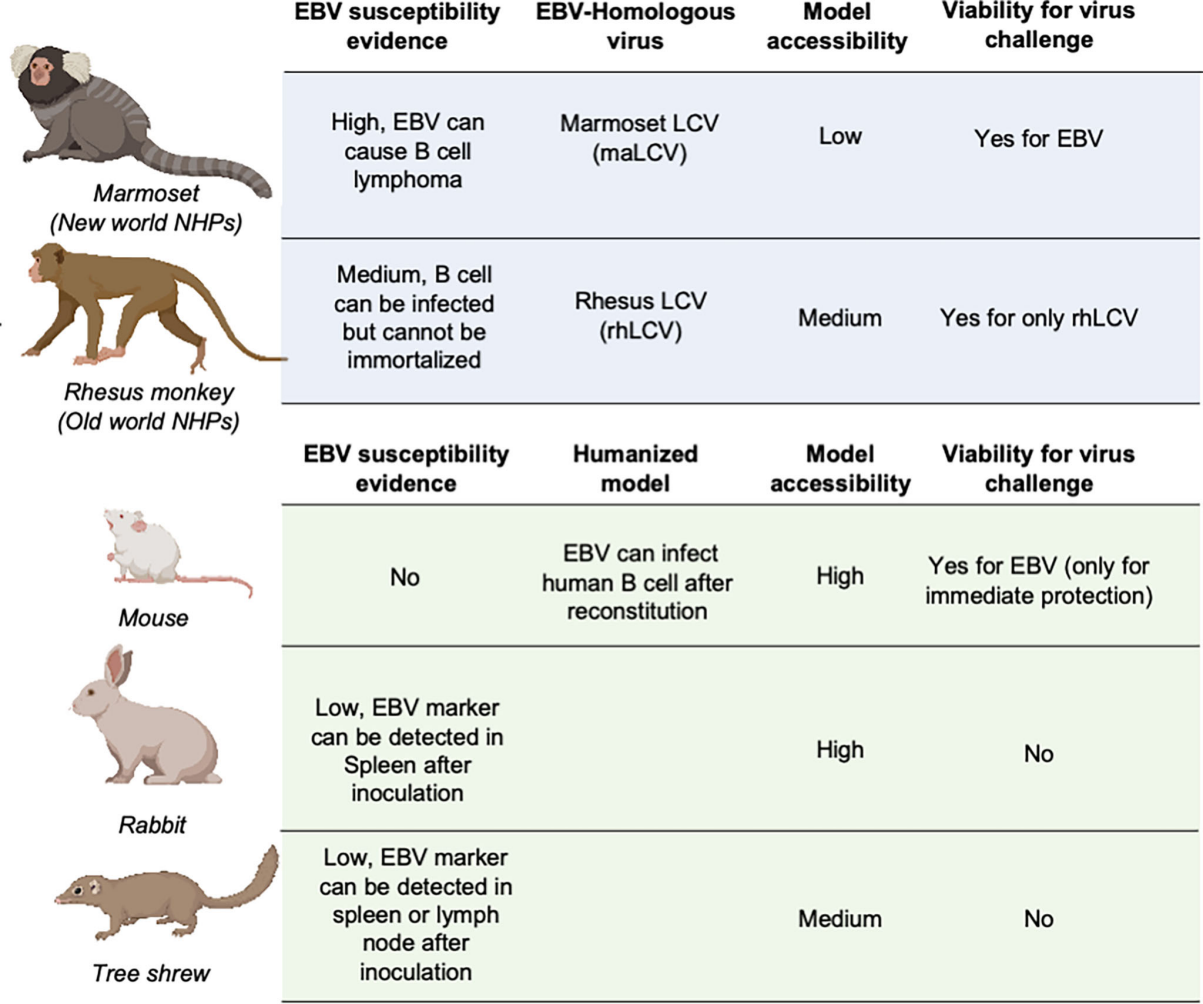
Animal models for EBV vaccine evaluation. Non-human primates are marked in light blue and other animal models are marked in light green. LCV, lymphocryptovirus.

Animal models are necessary and critical for the evaluation of infection or protection status against infectious disease pathogens and developing therapeutic drugs or vaccines. During the evaluation of vaccines against most pathogens, challenge experiments in animal models are considered the gold standard for the final assessment of vaccine efficacy (137–141). However, due to the restricted host tropism of EBV, a human herpesvirus, there is a limited range of susceptible candidate animal models (142–144) (**Figure 3**).

#### Non-Human Primates

The great similarity between humans and non-human primates (NHPs) encouraged the use of NHPs as challenge models for EBV vaccine evaluation (145). The fact that New- and Old-World NHPs are naturally infected by EBV-related herpesviruses or lymphocryptoviruses (LCVs) further demonstrated the potential value of NHP in EBV vaccine evaluation.

In the late 20^th^ century, the discoverer of EBV, Epstein et al. as well as Emini et al. used cotton-top tamarins and common marmosets (*Callithrix jacchus*) for gp350-based vaccine evaluation of both neutralizing antibody titers and challenge protection (146, 147). However, cotton-top tamarins are no longer a viable NHP model because of their critically endangered status, and the common marmoset is also listed on the IUCN Red List, which basically rules out these two NHPs from general use in EBV vaccine evaluation (148).

By contrast, rhesus macaque, as one of the Old World NHPs, has enjoyed broad use as an animal model for a variety of human viral infections, mostly due to its relatively larger population and successful artificial breeding. Although it is susceptible to its species-specific LCV (rhLCV), which shares a high level of genomic sequence similarity with EBV (149), EBV cannot stably infect and immortalize the B cells of rhesus macaques (150), which restricts the use of this animal model in challenge experiments. Therefore, the majority of EBV immunization studies used rhesus macaques as the animal model for evaluation of specific T cell responses (45, 151–153). And thus, instead of using EBV as challenge virus, rhLCV could be used as an equivalent virus for determining the immune protection from EBV infection. Singh et al. evaluated the protection efficacy of the anti-EBV gHgL neutralizing antibody AMMO1 *via* rhLCV challenge in rhesus macaques, and the protected animals showed higher plasma EBV neutralizing activity.

#### Other Mammalian Models

As the most widely used animal model, mice play an important role in EBV vaccine evaluation. Most prophylactic EBV vaccine studies used mouse immunization to primarily assess the serum antibody titer and neutralizing antibody titer (43, 44, 46). However, because mice cannot be naturally infected with EBV, humanized mice are used as an alternative animal model for EBV challenge experiments (37, 48, 54). These chimeric animals are constructed by transferring human CD34-positive hemopoietic stem cells into immunocompromised mice (154, 155). This model is appropriate for evaluating the efficacy of therapeutic treatment for immediate EBV challenge, rather than the eliciting of an adaptive immune response by a prophylactic EBV vaccine, since the mice have an incomplete immune system even after reconstitution and lack human epithelial cells. A humanized mouse model was also used to evaluate the protective efficacy of AMMO1 (63), and the results showed that the AMMO1 antibody could inhibit EBV infection.

Some studies also used rabbits as animal models for EBV vaccine evaluation (22, 27, 28, 51, 56, 155, 156). The anti-EBV VCA titer and EBV DNA level could be detected in the blood of most rabbits after intravenous, intranasal, or peroral inoculation. However, only a portion of rabbits showed positive EBERs, LMP1, or EBNA in splenectomized samples, and even fewer rabbits displayed sustained EBV positivity, accompanied by a heterogenous host reaction (157, 158). The uncertainty of the infection status hindered the use of rabbits as a challenge model, and most research studies only used rabbits as an immunization model for serum response evaluation (159).

Recently, it was found that the Chinese tree shrew (*Tupaia belangeri* subsp. *chinensis*) could also be a viable animal model for EBV vaccine evaluation. Following intravenous injection of virus, 8/10 tree shrews displayed symptoms of EBV infection including detectable expression of EBV-related genes and increase of anti-EBV antibodies. Despite positive results in early challenge, only a small portion of tree shrews showed EBER, LMP, and EBNA2-positive cells in spleen or mesenteric lymph node samples. The negative staining for EBV markers in the lungs and nasopharynx also indicated that epithelial cell infection might also be absent in the tree shrew animal model (160, 161).

### Assessment of Immune Protection Efficacy

After confirming the design of a vaccine and immunization methods, assessment of immune protection efficacy would be critical for vaccine evaluation (162). For prophylactic vaccines against infectious pathogens, the key index revealing the efficacy of immunization protection is the neutralizing antibody (nAb) titer (163, 164), since neutralizing antibodies can efficiently block the virus from interacting with the host receptor, preventing viral attachment and membrane fusion. Therefore, a higher anti-EBV neutralizing antibody titer indicates better protection against EBV infection and could theoretically also reduce the incidence of EBV-associated malignancies. Although the presence of neutralizing antibodies is theoretically sufficient evidence for protection against viral infection, the value of this index in predicting the incidence of malignancies remained unclear (165, 166). A large cohort study conducted in Taiwan (167) indicated that EBV B cell neutralization capability of the serum or the anti-gp350 antibody titer was associated with lower risk of nasopharyngeal carcinoma. However, Zhu et al. recently performed a prospective cohort study on EBV glycoprotein-targeting neutralizing antibody titers in plasma samples from nasopharyngeal carcinoma (NPC) patients and healthy controls, which revealed that there was no significant difference in neutralizing antibody titers against EBV glycoproteins, including gp350, gHgL, gp42, and gB (168).

During the evaluation of immune reaction against EBV, the T cell response is also considered critical part, especially for eliminating latent infection and adaptive immune responses against EBV-associated tumors (58, 60–62, 169, 170). A review concluded that T cell responses participate in the control of EBV in all phases of infection (171, 172). However, the majority of vaccine studies evaluating the T cell response were based on latent-phase proteins such as LMP and EBNA, while studies on T cell responses induced by EBV glycoproteins or T cell epitope mapping for glycoproteins were relatively rare. Thus, further studies on the T cell response elicited by EBV glycoproteins for controlling both primary infection and regulating immunological surveillance against EBV-associated malignant diseases could provide guidance for improving the evaluation systems for the assessment of prophylactic vaccine efficacy.

## Conclusion and Prospects

In recent years, prophylactic vaccines against EBV received significant attention, since the latest achievements in fundamental virology, vaccine technology and synthetic biology have brought new opportunities for vaccine development.

Early research studies on gp350 as a vaccine candidate revealed intrinsic shortage of gp350 in eliciting sufficient humoral immunity against primary infection. But still these studies become the forerunner for exploration of EBV glycoproteins as vaccine candidates. Recent progress in the discovery of epithelial cell receptors and elucidation of the infection mechanism of EBV highlights the critical function of gH/gL and gB during virus–host interaction and membrane fusion, indicating that they could be ideal major vaccine target for eliciting robust neutralizing antibody. Besides glycoproteins, although immunization with lytic and latent phase proteins is not able to provide protection against primary infection, the strong T cell immune response elicited by these proteins benefits the establishment of lasting immune surveillance of EBV latent infection and reinforcement of anti-EBV immunity after primary humoral defense. Additionally, an appropriate vaccine platform can improve the immunogenicity of certain antigens and enhance immune recognition. The adoption of protein modification *via* multimerization or fusion with immune cell-targeting domains may provide more possibilities for protein-based vaccines, while the application of synthetic nucleic acids as delivery systems could be the next milestone in the evolution of general vaccine design for not only EBV but all pathogens. Beyond vaccine design, a finer system for the evaluation of vaccine efficacy is also crucial for the development of a successful vaccine. A suitable animal model for EBV challenge is required. Further studies on the EBV-susceptibility of non-NHP models or viable NHP models would be as important as the innovation in EBV vaccine design. And it remains unclear whether T cell responses should be listed in the assessment system for determining the protection efficacy of EBV prophylactic vaccines, urging more intensive research on the connection between elicited cellular immunity and protection from both primary infection and malignancies.

Prospectively, with the advancement in understanding of immunity against EBV infection, more vaccine targets would be discovered, and using combinatorial antigens as vaccine candidate may display even promising immunization efficacy. The emerging vaccine platforms such as nanoparticle or mRNA may enjoy a broader application in development of EBV vaccines. And further studies on searching better animal models and evaluation indicators for assessment of EBV vaccine are required to assist the validation of protection efficacy after immunization.

## Author Contributions

CS wrote the original manuscript and generated the figures. XC generated the summary table of animal trials and clinical trials. YK and MZ provided guidance and reviewed the final manuscript. All authors contributed to the article and approved the submitted version.

## Funding

This study was supported by the National Natural Science Foundation of China (81801645, 82030046).

## Conflict of Interest

The authors declare that the research was conducted in the absence of any commercial or financial relationships that could be construed as a potential conflict of interest.

The handling Editor declared a past co-authorship with one of the authors MSZ.
